# Migration of pre-induced human peripheral blood mononuclear cells from the transplanted to contralateral eye in mice

**DOI:** 10.1186/s13287-021-02180-5

**Published:** 2021-03-10

**Authors:** Jianfa Huang, Bikun Xian, Yuting Peng, Baozhu Zeng, Weihua Li, Zhiquan Li, Yaojue Xie, Minglei Zhao, Hening Zhang, Minyi Zhou, Huan Yu, Peixin Wu, Xing Liu, Bing Huang

**Affiliations:** 1 State Key Laboratory of Ophthalmology, Zhongshan Ophthalmic Center, Sun Yat-sen University, Guangzhou, 510060 China; 2 The Second People’s Hospital of Foshan, Foshan, 528000 Guangdong China; 3 Guangdong Provincial Hospital of Traditional Chinese Medicine, Guangzhou, 510120 China

**Keywords:** Human peripheral blood mononuclear cells, Stem cells, Cell therapy, Migration, Ocular diseases

## Abstract

**Background:**

Retina diseases may lead to blindness as they often afflict both eyes. Stem cell transplantation into the affected eye(s) is a promising therapeutic strategy for certain retinal diseases. Human peripheral blood mononuclear cells (hPBMCs) are a good source of stem cells, but it is unclear whether pre-induced hPBMCs can migrate from the injected eye to the contralateral eye for bilateral treatment. We examine the possibility of bilateral cell transplantation from unilateral cell injection.

**Methods:**

One hundred and sixty-one 3-month-old retinal degeneration 1 (rd1) mice were divided randomly into 3 groups: an untreated group (*n* = 45), a control group receiving serum-free Dulbecco’s modified Eagle’s medium (DMEM) injection into the right subretina (*n* = 45), and a treatment group receiving injection of pre-induced hPBMCs into the right subretina (*n* = 71). Both eyes were examined by full-field electroretinogram (ERG), immunofluorescence, flow cytometry, and quantitative real-time polymerase chain reaction (qRT-PCR) at 1 and 3 months post-injection.

**Results:**

At both 1 and 3 months post-injection, labeled pre-induced hPBMCs were observed in the retinal inner nuclear layer of the contralateral (left untreated) eye as well as the treated eye as evidenced by immunofluorescence staining for a human antigen. Flow cytometry of fluorescently label cells and qRT-PCR of hPBMCs genes confirmed that transplanted hPBMCs migrated from the treated to the contralateral untreated eye and remained viable for up to 3 months. Further, full-field ERG showed clear light-evoked a and b waves in both treated and untreated eyes at 3 months post-transplantation. Labeled pre-induced hPBMCs were also observed in the contralateral optic nerve but not in the blood circulation, suggesting migration via the optic chiasm.

**Conclusion:**

It may be possible to treat binocular eye diseases by unilateral stem cell injection.

**Graphical abstract:**

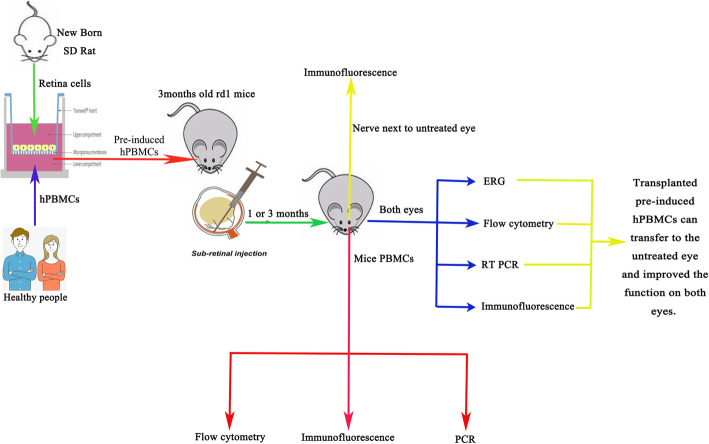

**Supplementary Information:**

The online version contains supplementary material available at 10.1186/s13287-021-02180-5.

## Background

Primary retinitis pigmentosa (RP) is a hereditary eye disease characterized by dystrophic degeneration of photoreceptor cells and retinal pigment epithelial (RPE) cells [1–7]. As RP often involves both eyes, it may lead to blindness and there are currently no effective treatments. Nerve growth factor therapy and gene therapy to preserve the viability and function of retinal cells are under study [8–10], but such treatments cannot restore visual function once these cells are lost. On the contrary, the transplantation of pluripotent stem cells with retinal cell differentiation capacity has the potential to reverse cell loss and improve the electrophysiological response to light.

It is reported that stem cells isolated from the adult mouse ciliary marginal zone can differentiate into specific retinal cells under appropriate culture conditions [11–13], while human and murine embryonic stem cells have been shown to differentiate into RPE cells as well as rhodopsin-expressing photoreceptor cells [14–16]. Transplantation of photoreceptor cell precursors into the subretinal space of mice with retinal degeneration also improved the electrophysiological response to light [17–19]. There are, however, many challenges to clinical application of stem cell therapy, including the isolation of stem cells able to differentiate along multiple retinal cell lineages and integrate into existing retinal circuits. Peripheral blood mononuclear cells (PBMCs) include a subpopulation of stem cells [20] showing multilineage differentiation potential under various induction conditions [21–25]. Further, these cells can be isolated relatively easily and in large quantities, making them a promising potential source for cell-based therapy research and clinical application [26–30]. Human PBMCs co-cultured in vitro with neonatal rat retinas and transplanted into the subretinal space of RP model mice (rd1 mice) can survive for at least 3 months, distribute to all retinal layers, and partially restore light-evoked electrophysiological responses [31–34], thus demonstrating potential utility for RP treatment.

The current stem cell therapy for ocular diseases is direct transplantation into the affected eye(s). If both eyes are diseased, it is assumed that transplantation must also be bilateral. However, transplanted cells may migrate from the treated eye to the contralateral eye via the circulatory system and/or optic chiasm [35–37]. This may allow unilateral stem cell transplantation for the treatment of bilateral eye diseases. To assess the potential of this strategy, we monitored the survival and migration of pre-induced hPBMCs from the injected eye to the untreated contralateral eye of rd1 mice.

## Materials and methods

### Experimental animals

This study was approved by the Medical Ethics Committee of Zhongshan Ophthalmic Center, Sun Yet-Sen University, Guangzhou, China (Protocol No. [2008] 15). All animal procedures were performed in accordance with the Association for Research in Vision and Ophthalmology (ARVO) Statement on the Use of Animals in Ophthalmic and Vision Research and approved by the Ethics Committee of Zhongshan Ophthalmic Center (Animal Welfare Assurance No. 2011-015). One hundred and sixty-one 3-month-old rd1 mice [38–40] were divided randomly into 3 groups. The untreated group of 45 rd1 mice received no treatment, the control group of 45 rd1 mice received right subretinal injection of serum-free DMEM, and the treatment group of 71 rd1 mice received right subretinal injection of pre-induced hPBMCs (Supplemental Table 1). Another six C57 mice were used as positive controls for full-field ERG detection. The retinas of eighteen 1-day-old Sprague Dawley (SD) rats were used to pre-induce hPBMCs. Three rats were used for each pre-induction trial. Rats were provided by the Experimental Animal Center, Sun Yet-Sen University (Animal Quality Certificates No.: 44008500009461, 44008500009006, 44008500005627, and 44008500005343).

### Isolation of hPBMCs

Two hPBMCs samples were collected from two healthy human donors (36 and 58 years old) and co-cultured with retina tissues of neonatal rats for 4 days before subretinal injection. Both donors provided informed consent prior to experiments. The hPBMCs were then isolated as described in Supplemental Method 1.

### Pre-induction of hPBMCs

The upper compartments of 6-well transwell plates were pre-seeded with rat retinal cells isolated as described (Supplemental Method 2) and maintained in neural stem cell culture medium. The lower chambers were then seeded with hPBMCs in suspension at 0.5 mL/well, and the plates placed in an incubator maintained at 37 °C with an atmosphere of 5% CO_2_ and 100% humidity for 4 days. Each day, changes in hPBMCs morphology were examined under a phase-contrast microscope (Leica, Wetzlar, Germany). A half volume of neural stem cell culture medium in the upper compartment was exchanged for fresh medium each day.

### Changes in hPBMCs morphology and marker expression phenotype during pre-induction culture

Changes in hPBMCs morphology and lineage marker expression profile during pre-induction culture with rat retinal tissue were examined by phase-contrast microscopy (Leica, Wetzlar, Germany) and qRT-PCR. The detailed methods for immunofluorescence staining and flow cytometry (Supplemental Table 2) can be found in our previous study [34] and Supplemental Methods.

### Subretinal transplantation of hPBMCs after pre-induction culture

Mice were anesthetized by intraperitoneal injection of pentobarbital sodium (60 mg/kg body weight). Right eyes were administered compound tropicamide eye drops for dilation, and superficially anesthetized with tetracaine hydrochloride eye drops. The sclera was exposed by blunt dissection of the conjunctiva. To facilitate subretinal injection, one small hole was produced by inserting a corneal needle at 2-mm behind the limbus. To prevent an elevation in intraocular pressure during subretinal injection, another small hole was produced at a 180° angle in front of the corneal limbus. The control group was injected with 2 μL of serum-free DMEM and the treatment group with 2 μL of cell suspension containing 5–6 × 10^5^ cells labeled with 1,1′-dioctadecyl-3,3,3′,3′-tetramethylindocarbocyanine(CM-DiI) in serum-free DMEM (Supplemental Method 3) via a microsyringe. Briefly, after the cornea was moistened with normal saline, microinjection was performed through the reserved needle hole with the needle inclined upward at 15°. Upon insertion into the subretinal space, the needle was stopped immediately, and the cell suspension injected slowly. The needle was withdrawn slowly after the cell suspension was completely injected. Local swelling and whitening of the retina were typically observed at the point of injection but without intraocular bleeding. After needle withdrawal, the injected eye was smeared with tobramycin eye ointment and treated with tobramycin eye drops three times daily for 1 week.

### Preparation of frozen retinal sections and immunofluorescence staining

At 2 weeks, 1 month, and 3 months post-injection, both eyes were extracted and examined by immunofluorescence (*n* = 3 mice each per time point in the control and untreated groups, and *n* = 5 mice per time point in the treatment group). Briefly, after sacrifice by cervical dislocation, both eyes were removed, embedded with optimal cutting temperature (OCT) compound in a specified direction, and frozen at − 20 °C. Each frozen eye was sectioned 10 times at 5-μm thickness and placed on 5 adhesive slides for drying in a fume hood.

Sections were fixed with 4% paraformaldehyde (Whiga Technology, GuangZhou, China) for 5 min, washed three times with phosphate buffered saline (PBS) (5 min per wash), treated with 0.1% Triton-100 (Whiga Technology) for 15 min, washed three times with PBS (5 min per wash), and blocked in goat serum (Boster Biological Technology, Wuhan, China, http://www.boster.com.cn) for 1 h at room temperature (RT). Sections were then cleared of excess serum and incubated with an antibody against human mitofusin-1 (D6E2S, Rabbit mAb #14739, 1:100, Cell Signaling Technology, Danvers, MA, USA, https://www.cst-c.com.cn) overnight at 4 °C. The next day, sections were washed three times with PBS (5 min per wash) and then incubated with anti-rabbit IgG Fab2 (Alexa Fluor 4884412S 1:500 Cell Signaling Technology) under darkness for 1 h at RT, washed three times with PBS (5 min per wash), counterstained with Hoechst 33342 (1:1000; Sigma-Aldrich, St. Louis, MO, USA) under darkness for 5 min, and then rinsed gently with PBS under darkness. Sections were cleared of excess PBS, dripped with anti-fade buffer medium, and mounted under coverslips. Slices were protected from light during these procedures and thereafter. Mouse PBMCs were isolated and detected as described in Supplemental Method 4. Stained sections were observed under a laser scanning confocal microscope (Carl Zeiss, LSM880, Oberkochen, Germany, http://www.zeiss.com) and photographed.

### Flow cytometry

At 1 and 3 months after subretinal injection, retinal cells were isolated from 6 mice in the untreated group, 6 mice in the control group, and 8 mice in the treatment group for counting of CM-DiI-stained and immunostained cells by flow cytometry. Briefly, after sacrifice by cervical dislocation, both eyes were removed and rinsed with PBS. The cornea, lens, and vitreous body were removed under a stereomicroscope and the retina isolated. In the untreated and control groups, the right retina was isolated from each mouse and randomly placed in a tube with another retina of the same group. In the treatment group, two retinas were placed in each tube but right (ipsilateral) and left (contralateral) retinas were kept separate. Retinas were rinsed three times with PBS, digested with 1 mL trypsin for 5 min, and triturated into a cell suspension. Cells were then centrifuged at 1500 rpm for 5 min and the supernatant discarded. Next, the pellet was rinsed and resuspended in PBS. This washing and centrifugation cycle was repeated two more times, and the final suspension passed through a 200-mesh filter. Cells were fixed in 4% paraformaldehyde at RT for 5 min, rinsed three times with PBS, permeablized with 0.1% Triton-X (Whiga Technology) at RT for 5 min, and rinsed three times with PBS. Cells were then incubated with FITC-labeled anti-human mitochondrial-specific antibody (rabbit anti-human mitochondrial antibody, Alexa Fluor® 488 1:50; Millipore, Burlington, MA, USA) at RT for 30 min. In preliminary experiments, this antibody showed no cross-reaction with mouse retinal cells. Cells were incubated and stained according to the antibody suppliers’ instructions. Stained cells were rinsed two times with PBS and counted by flow cytometry (BD Bioscience, Franklin Lakes, NJ, USA, https://www.bd.com/). Mouse PBMCs were stained and analyzed by flow cytometry using the same methods.

### Electroretinography

Both eyes in the untreated group (*n* = 10), the control group (*n* = 10), treatment group (1 month: *n* = 30, 3 months: *n* = 20), and positive control group (*n* = 6) were examined by full-field ERG [41–43]. We reduced the influence of laboratory environment and operator by having the same experimenter complete all measurements on a single group of mice within the same day, and by reducing the movement of nearby people during the ERG examination. Mice were first dark-adapted for 12 h and anesthetized as described. Both eyes were then dilated and treated with moisturizing eye gel. Mice were placed on an insulation board in the right position with a reference electrode inserted in the cheek skin on the side of the measured eye and a ground electrode in the tail. After adjusting animal position to maximize corneal contact with the recording electrode, the eye was stimulated simultaneously for 2 ms with both ultraviolet light (365 nm) at 1.6 log(Cd s/m^2^) and green light (505 nm) at 1.3 log(Cd s/m^2^), and the stimulation waveform recorded by the Phoenix Ganzfeld full-field ERG (Phoenix Research Laboratories Inc., Pleasanton, CA, USA, http://www.phoenixreslabs.com).

### Measurement of human gene expression in mouse retina by quantitative reverse transcription-polymerase chain reaction (qRT-PCR)

At 1 and 3 months after subretinal injection, expression of marker genes was examined in 6 untreated, 6 control, and 8 treatment group mice. Briefly, after sacrifice by cervical dislocation, both eyes were removed and rinsed with PBS. The cornea, lens, and vitreous body were removed under a stereomicroscope, and the retina was isolated. In the untreated and control groups, right retinas were paired randomly in the same tube, while in the treatment group, right and left retinas were kept separate and placed two each in tubes. Retinas were rinsed three times with PBS, lysed in 1 mL TRIzol, and homogenized by trituration for 1 min. The lysate was incubated at RT for 5 min and centrifuged at 12,000*g* for 10 min at 4 °C. The supernatant was transferred to a new tube, mixed with chloroform, and shaken intensely for 2 min. The mixture was centrifuged at 12,000*g* for 10 min at 4 °C, and the upper layer (about 600 μL) transferred to a new tube with 600 μL isopropanol. The new mixture was shaken thoroughly, incubated at RT for 10 min, and centrifuged at 12,000*g* for 10 min at 4 °C. The supernatant was removed, mixed with 1 mL 75% alcohol, shaken gently, and centrifuged at 8000*g* for 5 min at 4 °C to precipitate the RNA. The RNA pellet was left at RT for about 5 min and then resuspended in 20 μL DEPC water. RNA quality and quantity were measured by spectrophotometry. The RNA was then reverse transcribed into cDNA using the PrimeSpript RT kit (RR047A, Takara http://www.takara.com.cn/) and the human-specific primer set f: GCTTGCAACTATAGCAACAGC and r: GGACTGTCTACTGAGTAGCC designed using Primer 5.0 software. Expression levels were then examined by qRT-PCR using TB Green™ Premix Ex Taq™ II (Tli RNaseH Plus, RR820A, Takara) and the StepOnePlus PCR system (Allied Biosystems, Foster City, CA, USA). The thermocycle protocol was 95 °C for 30 s followed by 40 cycles of 95 °C for 5 s and 60 °C for 30 s. The human GAPDH gene was used as the internal control (100 bp product).

### Measurement of mouse PBMC gene expression by qRT-PCR

RNA extraction and reverse transcription into cDNA were conducted as described above for mouse retina. Reactions containing 50 ng of cDNA were subjected to qRT-PCR using 2 × Rapid Taq Master Mix (P222, Vazyme Biotech, Nanjing, China), the human-specific primers described above, and an ABI2720 Thermal Cycler (Allied Biosystems). The thermocyle was 95 °C for 3 min, followed by 35 cycles of 95 °C for 15 s, 58 °C for 15 s, 72 °C for 15 s, and a final extension at 72 °C for 5 min. The PCR product was analyzed by 2% agarose gel electrophoresis.

The human GAPDH gene served as the internal control.

### Statistical analysis

All data are expressed as mean ± standard deviation (SD). Group means were compared by one-way ANOVA using SPSS 18.0 software. A *P* < 0.05 (two-tailed) was considered statistically significant for all tests.

## Results

### Changes in hPBMCs and neonatal rat retinal cells during pre-induction culture

Freshly isolated hPBMCs were round, phase-bright, and evenly suspended in the culture medium (Supplemental Figure 1A). On day 1 of pre-induction culture in transwell plates with rat retina (Supplemental Figure 1B), most hPBMCs were still round and suspended in the culture medium, but a few formed clusters or adhered to the culture well wall and formed one or more small synapses. On day 3 of pre-induction culture (Supplemental Figure 1C), more hPBMCs adhered to the wall and exhibited irregular morphology. After 4 days, hPBMCs were roughly classified into four categories: round cells, cells with one to three processes, cells aggregated in clusters, and large yellow cells (Supplemental Figure 1D-G). The total number of hPBMCs after 4 days of pre-induction culture was about 2/3 that of the initial number (Supplemental Table 3). The vast majority of hPBMCs exposed to CM-DiI prior to injection were fluorescent as determined by flow cytometry (92.33% ± 6.40%) (Supplemental Figure 2 and Supplemental Table 4). Up to 99% of cells were labeled with anti-human mitochondrial-specific antibody as measured by flow cytometry (Supplemental Figure 3).

During 4 days of pre-induction culture, the neonatal rat retinal cells transformed from small adherent clusters to layers covering the entire upper compartment of the 6-well transwell plate (Supplemental Figure 4).

**Fig. 1:**
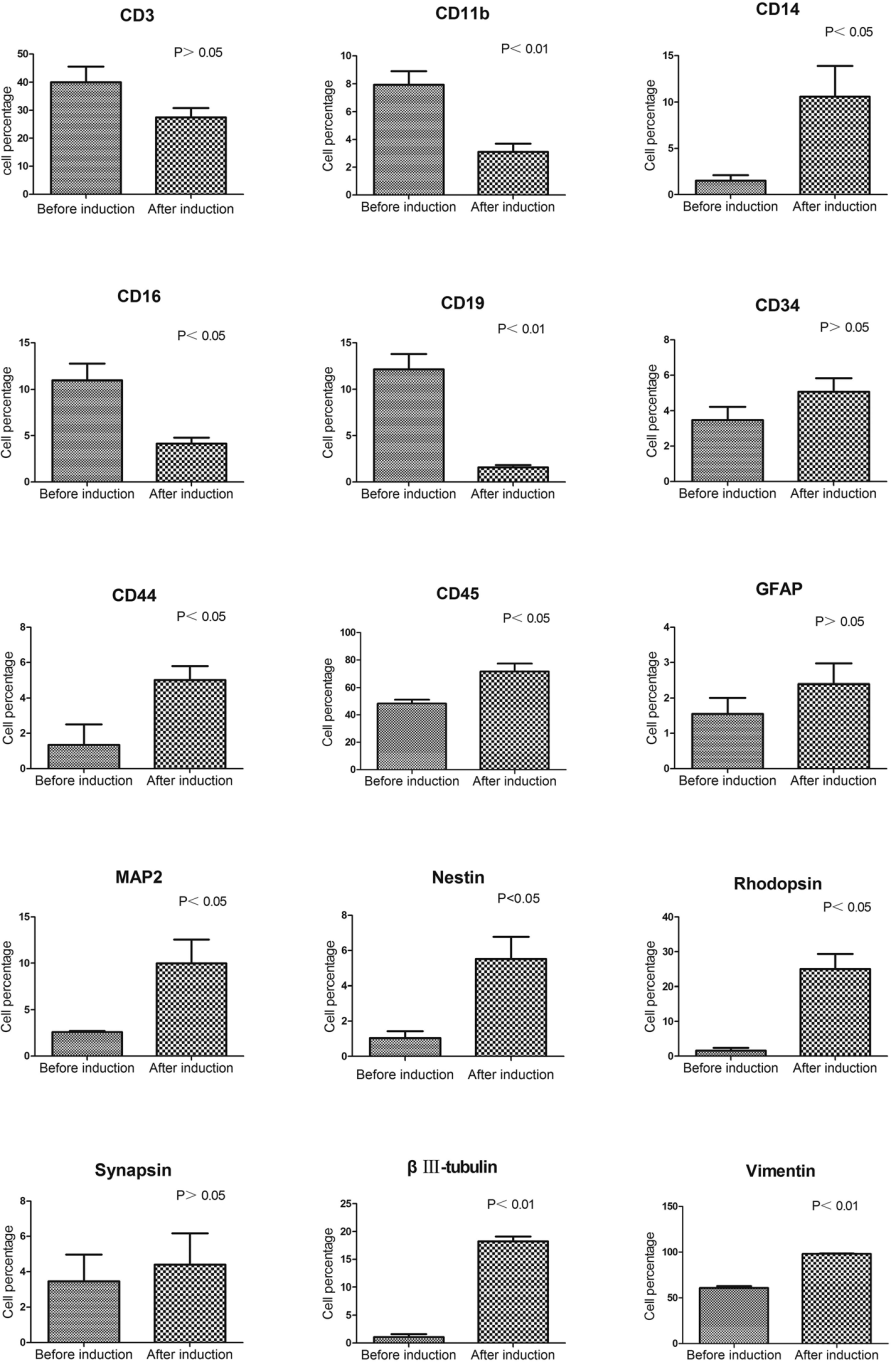
Changes in hPBMC phenotype during pre-induction culture with rat retina. Flow cytometry results showing changes in hPBMCs and retinal cell marker expression profiles during pre-induction culture. Expression levels of the retinal cell markers MAP2, nestin, rhodopsin, vimentin, beta III-tubulin, CD45, and CD14 increased significantly, while expression levels of the hPBMCs markers CD11b, CD19, and CD16 decreased significantly during culture. *P* < 0 .05 by paired sample t-test

Flow cytometry (Fig. 1 and Supplemental Table 5) and immunofluorescence staining (Supplemental Figure 5) indicated that the hPBMC lineage marker expression profile shifted during 4 days in pre-induction culture from predominantly blood lineage markers (CD11b, CD19, and CD16) to neural and photoreceptor lineage markers, including glial fibrillary acidic protein (GFAP), nestin, microtubule-associated protein 2 (MAP-2), beta-III tubulin, synapsin, and rhodopsin. Analysis by paired *t* test confirmed significantly increased expression levels of nestin, MAP-2, beta-III tubulin, and rhodopsin on day 4 (all *p* < 0.05). Further, qRT-PCR results were consistent with immunofluorescence staining and flow cytometry [34]. These findings suggest that pre-induction culture can drive hPBMC differentiation toward neuronal and photoreceptor lineages.

### Pre-induced hPBMCs can survive at least 3 months post-transplantation and migrate to the contralateral eye

**Fig. 2:**
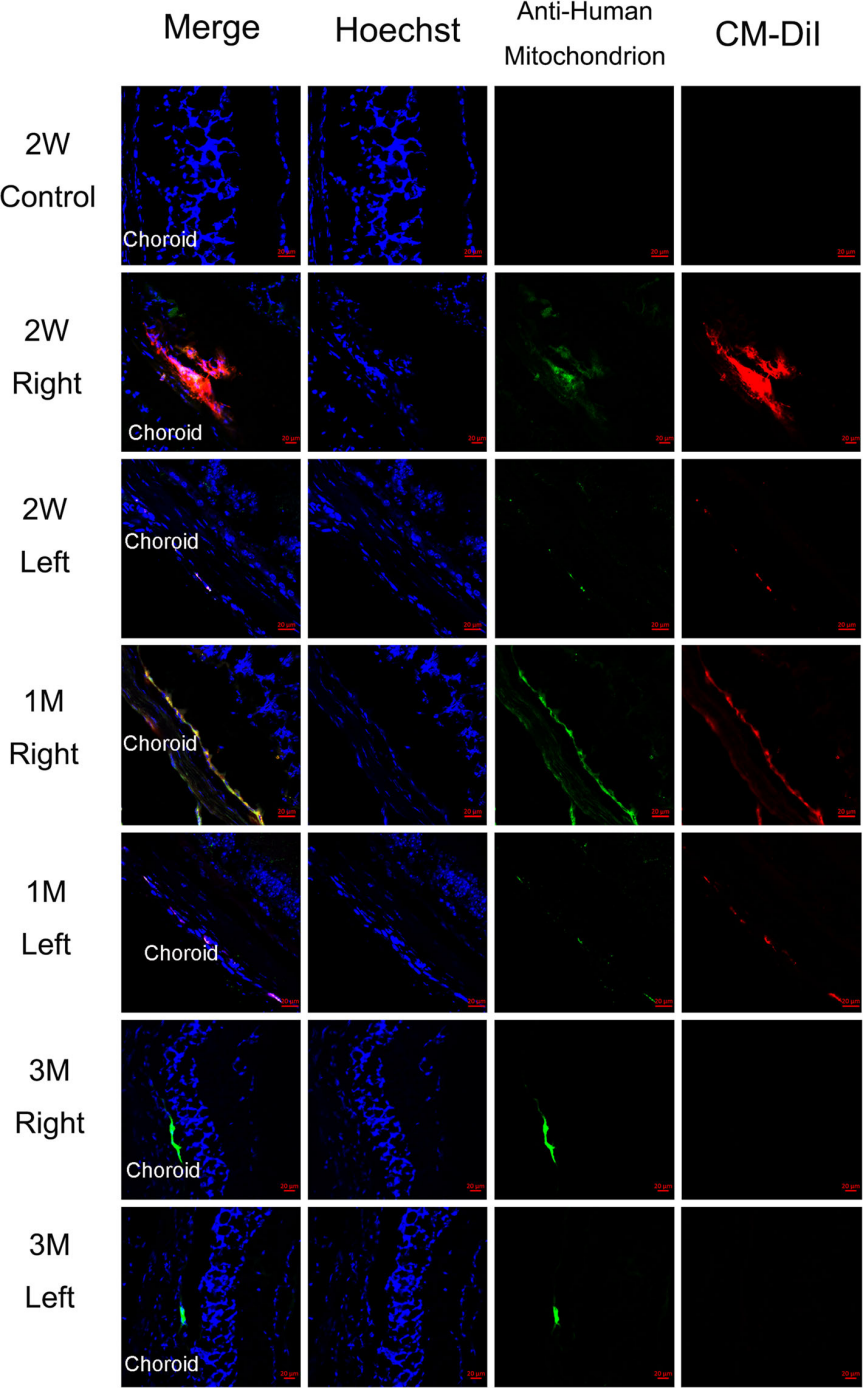
Immunofluorescent staining of frozen sections from rd1 mouse eyes following unilateral injection of pre-induced hPBMCs. Cells were labeled with CM-DiI (red) before injection and with human mitochondrial antibody ex vivo (green). At 2 weeks and 1 month after injection, double-labeled cells were clearly observed in sections from both the injected (right) retina and the contralateral (left) retina. Many fewer double-labeled cells were observed in both eyes at 3 months post-injection. The photos are all 400 ×

We labeled pre-induced hPBMCs with CM-DiI (red) prior to injection and with anti-human mitochondrial antibody (green) ex vivo at various times post-injection to track survival and migration (Fig. 2). No double-labeled cells were found 2 weeks, 1 month, or 3 months post-injection in ipsilateral (right) and contralateral (left) retinal slices from the untreated group (Supplemental Figure 6) and control group (Supplemental Figure 7). As expected, however, nuclear counterstaining (blue) revealed progressive degeneration of retinal cells in these rd1 mice [44, 45]. In the treatment group, double-labeled hPBMCs were clearly observed in the inner nuclear layer (INL) of the treated (right) eye at 2 weeks post-injection, while very few were found in the contralateral (left) eye. One month after transplantation, there were fewer hPBMCs in the INL of the treated eye but a relatively greater number in the contralateral eye compared to 2 weeks post-injection. At 3 months post-injection, there were significantly fewer double-labeled cells in both retinas compared to 1 month post-injection.

**Fig. 3:**
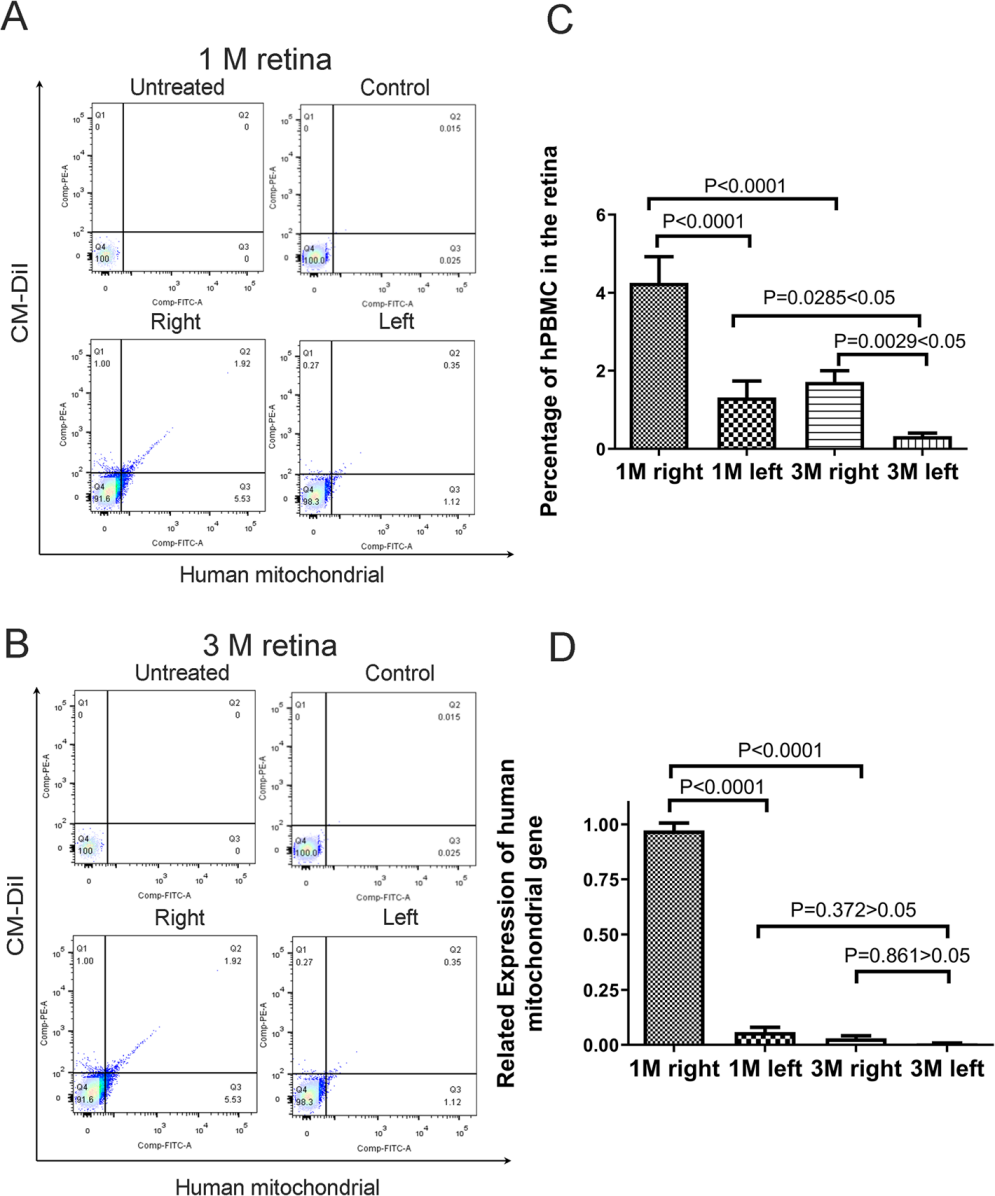
Analysis of surviving hPBMCs in the injected right retina and contralateral left retina by flow cytometry and real-time PCR. **a**–**c** Flow cytometry results showing surviving hPBMCs in retinal cell suspensions prepared **a** 1 month after transplantation and **b** 3 months after transplantation. The PE fluorescence (red) is from CM-DiI and the FITC fluorescence (green) is from immunostaining using a human mitochondrial antibody. Zone Q2 is the double-labeled area indicative of transplanted hPBMCs. **c** Statistical analysis of surviving hPBMCs in right and left retinas at 1 and 3 months after transplantation. **d** RNA expression of the human mitochondrial marker gene (indicative of hPBMCs). Expression level is shown relative to that at 1 month post-injection. *P* < 0 .05 by paired sample *t* tests

To verify and quantify migration of hPBMCs from the injected to contralateral eye, we conducted flow cytometry and qRT-PCR. Flow cytometry of retinal cell suspensions using fluorescent markers of the injected hPBMCs (a tagged human mitochondrial antibody and CM-DiI) revealed labeled cells in both injected and contralateral eyes at 1 month post-injection (Fig. 3a and c, Supplemental Figure 8) with greater numbers in the injected (right) eye compared to the left eye (4.255% ± 0.6662% vs. 1.313% ± 0.4267%). Consistent with analysis of stained retinal sections (Fig. 2), the numbers of surviving hPBMCs were reduced in both ipsilateral and contralateral eyes at 3 months post-injection (1.710% ± 0.2905% vs. 0.3275% ± 0.0793) (Fig. 3b and c, Supplemental Figure 9). Further, qRT-PCR demonstrated human mitochondrial gene expression in both eyes at 1 month post-injection, consistent with flow cytometry (Fig. 3d).

### Transplantation of pre-induced hPBMCs into one eye can improve the function of both eyes

**Fig. 4:**
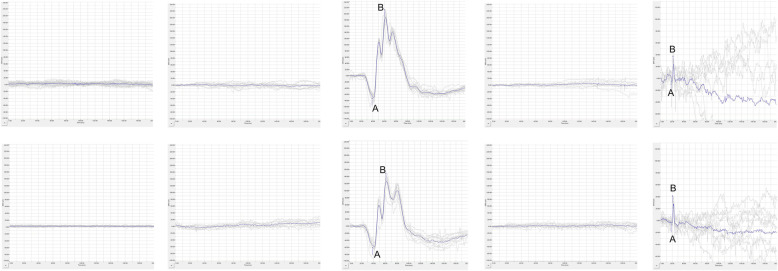
Improvement of light-evoked electroretinogram (ERG) signals by hPBMC injection. Each eye was stimulated simultaneously for 2 ms with ultraviolet light (365 nm) at 1.6 log(Cd s/m^2^) and green light (505 nm) at 1.3 log(Cd s/m^2^). Light-evoked ERG responses as observed in wild type C57 mice (*n* = 6) (middle panels) were absent or markedly smaller in untreated (*n* = 10) and control group rd1 mice (*n* = 10) (left panels). Response was also largely absent in treatment group mice at 1 month (*n* = 30) post-injection but both a and b waves were relatively large at 3 months (*n* = 20) post-injection (right panel)

To examine whether these transplanted pre-induced hPBMCs actually improved eye function, we compared the full-field ERG among groups at 1 and 3 months post-injection (Fig. 4). Light-evoked ERG waveforms were easily measured in wild type C57 mice (a-wave: − 63.13 ± 30.65 μV; b-wave: 203.4 ± 89.1 μV) (Supplemental Figure 10), but were absent or barely above noise level in right and left eyes of untreated and control group rd1 mice. In the treatment group, there was also little or no light response at 1 month post-injection, but a and b waves were evoked by light in both eyes at 3 months post-injection (a-wave: right eyes = − 3.71 ± 3.56 μV, left eyes = − 2.83 ± 2.68 μV; b-wave: right eyes = 25.67 ± 12.53 μV, left eyes = 21.47 ± 13.19 μV) (Supplemental Figure 11). There were no statistical differences between the untreated eyes and treated eyes after 3 months (a-wave: *P* = 0.155, b-wave: *P* = 0.085), while statistical differences were detected between C57 and rd1 mice 3 months after injection (*P* < 0.05). These findings are consistent with migration of hPBMCs to the contralateral eye and induction of retinal cell (light-responsive) phenotypes.

### Transplanted pre-induced hPBMCs did not migrate to the contralateral eye via the blood circulation

**Fig. 5:**
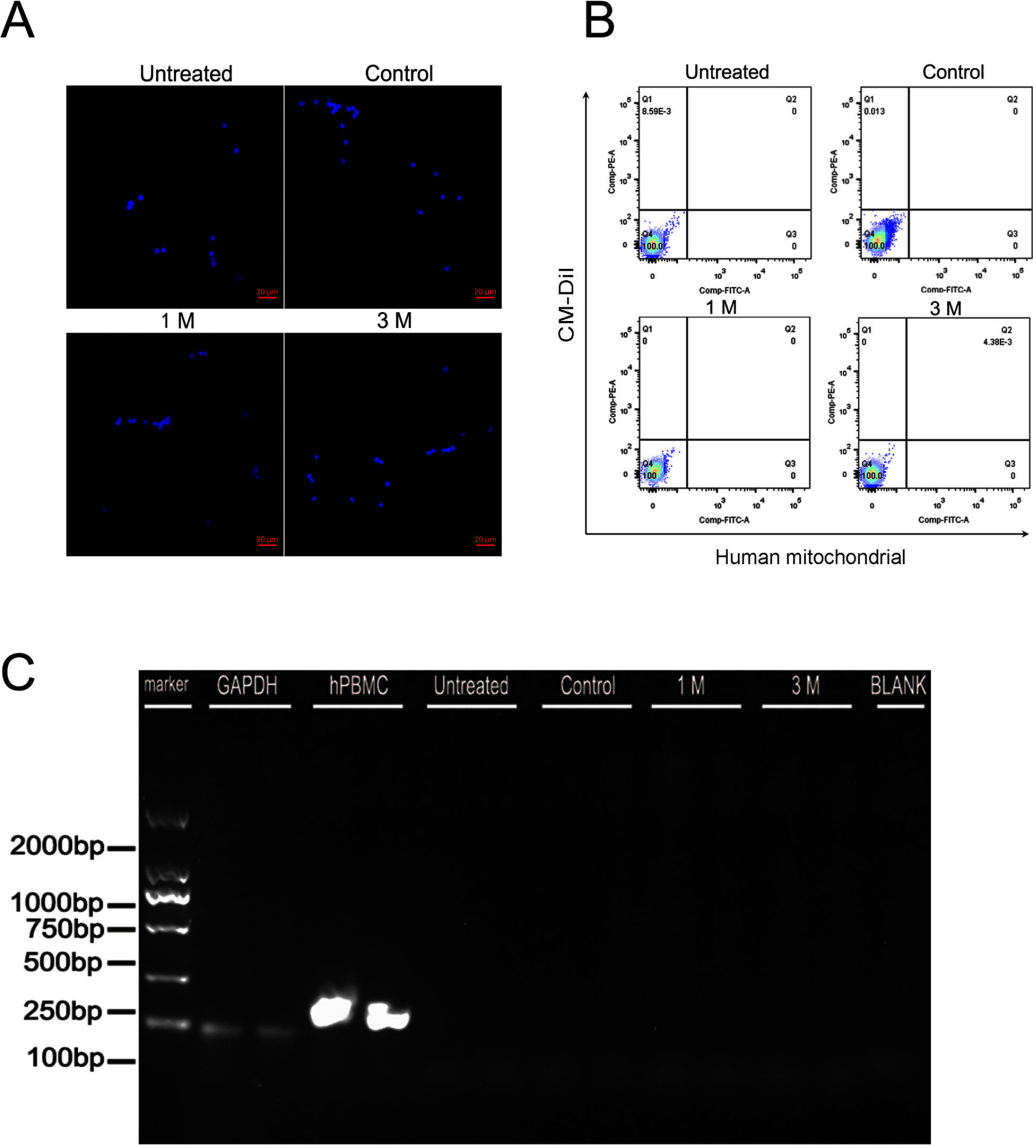
Absence of hPBMCs in the circulation of injected mice. **a** The hPBMCs of injected mice showed only Hoechst staining (blue) but no CM-DiI staining (red) or human mitochondrial antibody immunostaining (green). **b** Flow cytometry of treatment group mouse PBMCs, again showing no CM-DiI/FITC double-stained cells (zone Q2). **c** RT-PCR showing no expression of the human mitochondrial marker gene among mouse PBMCs at one and 3 months post-injection

There are two potential migration routes for injected hPBMCs from the injected eye to the contralateral eye, via the blood circulation and through the optic chiasm. There is a blood–ocular barrier in vivo, but this may be breached during subretinal injection, allowing entry of hPBMCs into the circulation and eventually into the contralateral eye. Alternatively, injected cells may migrate along the ipsilateral optic nerve to the contralateral optic nerve via the optic chiasm. No labeled cells or marker genes were found among the circulating PBMCs of injected mice by flow cytometry (Fig. 5b), RT-PCR (Fig. 5c), or immunofluorescence staining (Fig. 5a, Supplemental Figure 12).

### Transplanted pre-induced hPBMCs may migrate to the contralateral eye via the optic chiasm

**Fig. 6:**
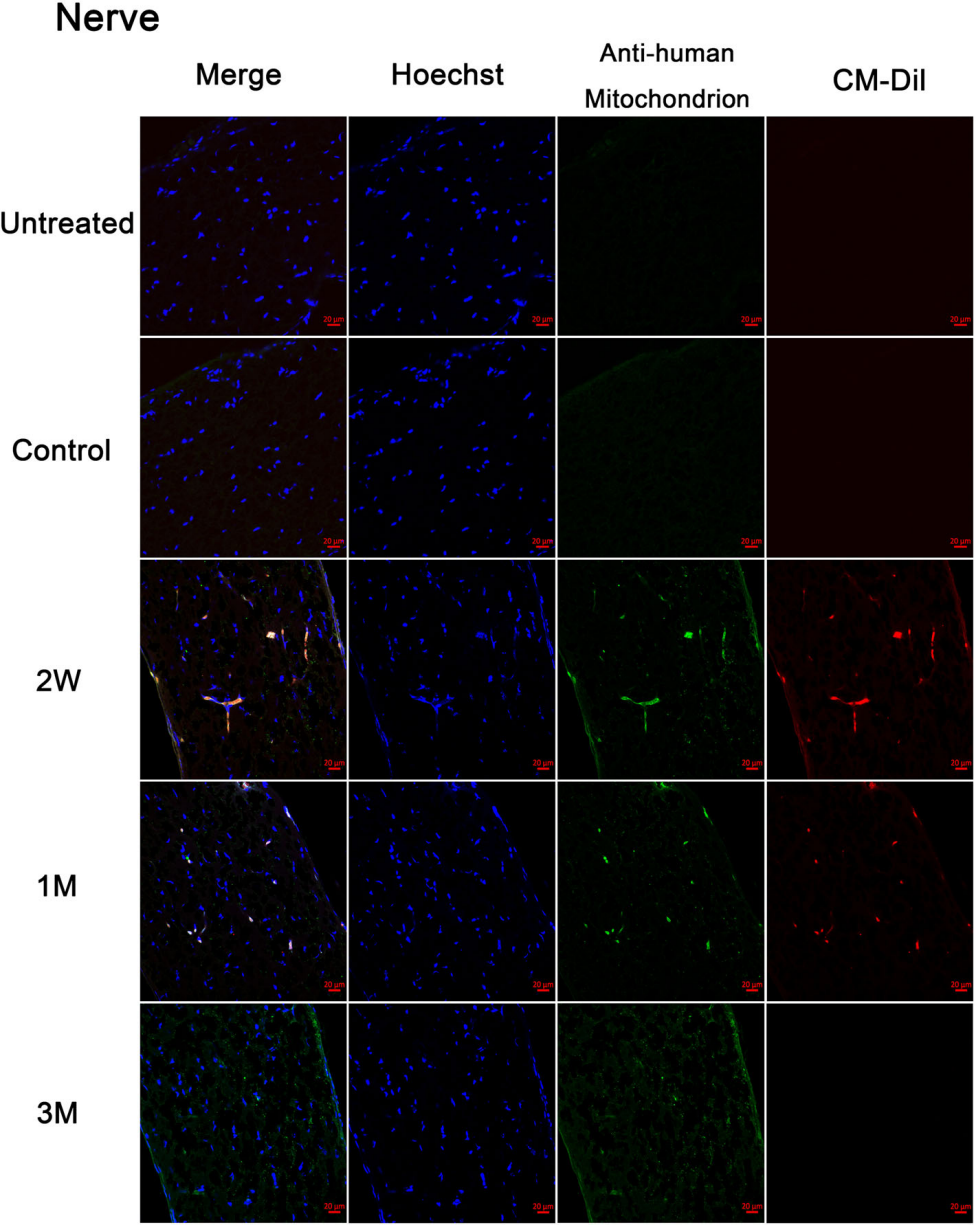
Migration of injected hPBMCs to the contralateral optic nerve. Frozen sections of the contralateral (left) optic nerve contained CM-DiI-stained cells (red) and human mitochondrial antibody-positive cells (green) at 2 weeks and 1 month after injection of CM-DiI-labeled hPBMCs into the right retina. All the photos are 400 ×

Since transplanted pre-induced hPBMCs were absent in the circulation, we examined the presence of labeled cells in frozen sections from the mouse optic nerve. No such cells were found in sections from untreated and control group mice, but were observed in the contralateral (left) optic nerve of treatment group mice at 2 weeks and 1 month post-injection. Few such cells were found at 3 months post-injection (Fig. 6).

## Discussion

Retinitis pigmentosa often involves both eyes and so may lead to blindness as the retina is not a self-repairing tissue [46–48] and there are currently no effective treatments. Replacement of lost cells, such as through stem cell treatment, is a potential strategy for improving functional loss from RP. For bilateral disease, this would presumably require transplantation into both affected eyes. However, we speculated that since the eyes are cross-connected by blood circulation and optic nerves [35–37], cells transplanted into one eye may migrate to the contralateral retina. If our hypothesis is true, injected cells from the more severely affected eye could migrate to the milder affected eye, thereby improving the function of both eyes. In this case, one less operation is required, reducing patient pain, distress, and the risk of adverse events, as well as accelerating the therapeutic schedule. Here we first demonstrated that the majority of hPBMCs survived during 4 days of pre-induction culture with rat retinal tissue, and many differentiated to express neuron- and photoreceptor-specific proteins. Further, these pre-induced hPBMCs survived for up to 3 months following injection into the subretinal space, where they further differentiated and integrated into functional circuits as evidenced by the reappearance of full-field ERG a and b waves in response to light. Surprisingly, these pre-induced hPBMCs were observed in both the treated right and untreated left eye 2 weeks, 1 month, and 3 months after cell injection as evidenced by CM-DiI and human antibody labeling. Transplanted cells were present not only in the retinal inner nuclear layer of the treated eye, but also in the retinal inner nuclear layer and optic nerve of the contralateral untreated eye. This migration was also confirmed by flow cytometry and qRT-PCR detection of transplanted cell markers. Moreover, the untreated eye also exhibited light-evoked a and b waves on the full-field ERG, implying that viable cells migrate via the optic chiasm and integrate into retinal circuits within the contralateral eye. Thus, unilateral injection of stem cells may induce recovery of visual function in the untreated eye of patients with bilateral retinal diseases.

Transplanted hPBMCs were observed in the contralateral eye of the treatment group, indicating that there is a transfer channel with the treated eye. The eyeball is wrapped by dense sclera [49–51] and the blood–retinal barrier (BRB) [52], so only blood vessels and optic nerves provide a potential connection between eyes [35–37]. However, we found hPBMCs in immunofluorescence sections of the contralateral optic nerve and excluded the possibility of a blood circulation pathway. Therefore, we speculate that hPBMCs may enter the contralateral eye through the optic nerve. In future research, we will use PCR and more precise tracing methods to verify this hypothesis.

The subretinal space was chosen as the graft site in this study because it is recognized as an immune privilege area [53–55]. Thus, if the BRB is intact, heterogonous cells injected into the subretinal space will not induce an immune reaction. However, cells entering the subretinal space will interact with an altered microenvironment in the diseased retina as evidenced by the promotion of cell differentiation and migration of transplanted cells not observed in intact retina [56–58]. In turn, transplanted cells can alter the microenvironment of the diseased retina to protect surviving cells and prevent further disease progression [59–64].

The most critical issues for the clinical efficacy of stem cell transplantation are in vivo survival, differentiation, integration, and phenotype stability. In this study, we demonstrated improvement of light-evoked ERG responses in both eyes at 3 months but not at 2 weeks and 1 month after transplantation, even in the treated eye, suggesting that injected cells can survive and integrate into functional circuits but that this process is relatively slow.

Further, survival time may have been underestimated by CM-DiI labeling. CM-DiI labels cells by binding to membrane lipids, and this labeling is maintained for multiple cell passages, allowing for the tracking of cell survival, proliferation, and migration over weeks and months. In our preliminary experiments, however, it was found that CM-DiI labeling was very weak at 3 months after cell transplantation. While this may indicate reduced survival, CM-DiI is quenched slowly after labeling according to the manufacturer. In addition, exposure to air for dual immunofluorescence staining of frozen sections may accelerate the quenching of the CM-DiI fluorescence. Indeed, we detected CM-DiI fluorescence by flow cytometry at 3 months after cell transplantation but few dual-stained cells were observed in frozen sections. Thus, the current method may not be optimal for detecting the long-term survival of injected cells and other techniques should be investigated.

These rd1 mice exhibit rapid retinal degeneration due to mutations in rod cell-specific cyclic guanosine monophosphate phosphodiesterase-6 (cGMP-6). The rod cells of rd1 mice began to degenerate 10 days after birth and are nearly or completely absent by 21 days after birth, while cone cells are absent by age 2–4 months. This rapid retinal degeneration model is therefore particularly convenient for experimental studies on retinopathy treatment and was used in the current study to examine the possibility of contralateral cell migration and ERG changes following unilateral hPBMC transplantation.

## Conclusion

We demonstrate that pre-induced hPBMCs transplanted unilaterally into the subretinal space of retinitis pigmentosa model mice can migrate to the contralateral untreated eye and survive for at least 3 months. Further, these cells appear to at least transiently integrate into functional retinal circuits. Finally, we demonstrate that pre-induced cells can migrate from the treated to contralateral untreated eye, likely via the optic chiasm rather than the blood circulation, although this notion requires further experimental verification. Collectively, these results suggest that cells can be transplanted into a single eye to treat both eyes, which may provide a simplified method for research on bilateral eye diseases using stem cells.

## Supplementary Information

**Additional file 1.**

## Data Availability

Data sharing is not applicable to this article as no datasets were generated or analyzed during the current study.

## Abbreviations

DMEM: Dulbecco’s modified Eagle’s medium
hPBMCs: Human peripheral blood mononuclear cells
ERG: Electroretinogram
qRT-PCR: Quantitative real-time polymerase chain reaction
RP: Retinitis pigmentosa
RPE cells: Retinal pigment epithelial cells
PBMCs: Peripheral blood mononuclear cells
rd1 mice: RP model mice
ARVO: Association for Research in Vision and Ophthalmology
SD rats: Sprague Dawley rats
CM-DiI: 1,1′-dioctadecyl-3,3,3′,3′-tetramethylindocarbocyanine
OCT compound: Optimal cutting temperature compound
PBS: Phosphate buffered saline
RT: Room temperature
RT-PCR: Reverse transcription-polymerase chain reaction
SD: Standard deviation
GFAP: Glial fibrillary acidic protein
MAP-2: Microtubule-associated protein 2
INL: Inner nuclear layer
BRB: Blood–retinal barrier
cGMP-6: Cyclic guanosine monophosphate phosphodiesterase-6
