# Supplementary material for: Migration of pre-induced human peripheral blood mononuclear cells from the transplanted to contralateral eye in mice
Source: Stem Cell Res Ther. 2021 Mar 10;12:168. doi: 10.1186/s13287-021-02180-5 (PMC7945672; doi:10.1186/s13287-021-02180-5)
Supplement: Supplementary file 1 — Additional file 1. [file 13287_2021_2180_MOESM1_ESM.doc]

**Supplemental information**

**Supplemental methods**

**1. Isolation of hPBMCs**

Blood samples (30 mL) were withdrawn from 2 healthy adult donors into prepared anticoagulant tubes containing heparin (Tianjin Biochemical Pharmaceutical Co. Ltd; China) and mixed 1:1 (v/v) with phosphate buffered saline (PBS) containing 1000 U/mL gentamicin (150 μL/100 mL）. The diluted blood was slowly added to 15−20 mL of lymphocyte separation medium (Haoyang Biotech, Tianjin, China, http://www.tbdscience.com) along the wall of a centrifuge tube at a ratio of 1:1, and then centrifuged at 2000 rpm for 20 min. The PBMC layer (the nepheloid layer between lymphocyte separation medium and plasma) was transferred carefully using a pipette to a new centrifuge tube and mixed with the same volume of PBS containing gentamicin, and the mixture centrifuged at 1600 rpm for 5 min. After centrifugation, the supernatant was removed. Then, 3.5 mL of red blood cell lysate (Beijing Leagene Biotechnology Co. Ltd; China) was added to the centrifuge tube. The mixture was triturated carefully for 5 min, topped up with an appropriate volume of PBS containing gentamicin, and centrifuged at 1600 rpm for 5 min. The supernatant was removed and the pellet resuspended in 20 mL of normal saline. Cell count was determined manually under a light microscope. After counting, the remaining suspension was centrifuged at 1600 rpm for 5 min, and the pellet resuspended in 3 mL of neural stem cell culture medium composed of DMEM/F12 (90 mL/100 mL; Gibco, Grand Island, NY, http: //www.invitrogen.com), human basic fibroblast growth factor (hbFGF; 10^3^ ng/100 mL; PeproTech, Rocky Hill, NJ, http://www.peprotech.com), human stem cell factor (hSCF; 200 ng/100 mL; PeproTech), human epidermal growth factor (hEGF; 10^3^ ng/100 mL; PeproTech), B27 stem cell culture supplement (50×; 2 mL/100 mL; Gibco), L-glutaminase (LG; 3%, 1 mL/100 mL; Whiga Technology, Guangzhou, China, http://whiga.biomart.cn), fetal bovine serum (FBS; 7 mL/100 mL; SiJiQing Biotech, Huzhou, China, http://www.hzsjq.com), and gentamicin (150 μL/100 mL; Tianxin Pharmaceuticals, Guangzhou, China, http://www.tianxin.com.cn) [34]. The cells were then seeded into the lower chambers of transwell plates also containing rat retinal tissue in the upper chambers as described in the main text.

**2. Acquisition and culture of retinal tissue from neonatal Sprague Dawley rats**

Neonatal Sprague Dawley (SD) rats were anesthetized at 4 °C. Their heads were removed, placed in sterile Petri dishes, and rinsed repeatedly with PBS containing 1000 U/mL gentamicin (150 μL/100 mL; Tianxin Pharmaceuticals, Guangzhou, China; http://www.tianxin.com.cn). Eyeballs were removed completely and the retina separated out from the other structures. Isolated retinas were rinsed in PBS containing gentamicin, torn with tweezers, and slowly triturated through a 1 mL syringe to produce a single cell suspension. The suspension was centrifuged and the pellet resuspended in 6 mL of neural stem cell culture medium The upper compartments of 6-well transwell plates (Catalog No. 3450; Corning Inc. Corning, NY, http://www.corning.com) were seeded with 1 mL of the SD retinal cell suspension supplemented with 1 mL of fresh neural stem cell culture medium, while each lower compartment was filled with 1.5 mL of fresh cell-free neural stem cell medium. The plates were placed in an incubator at 37 °C under 5% CO_2_ and 100% humidity overnight for standby use. The lower chambers were then seeded with hPBMCs as describe in the main text.

**3. Labeling of hPBMCs with CM-DiI**

After 4 days of pre-induction culture, rat retinal cells in the upper compartments were removed and discarded while hPBMCs in the lower compartments were harvested and centrifuged at 1500 rpm for 5 min. After removal of the supernatant, cells were rinsed with PBS, centrifuged once again, and resuspended in serum-free medium at 1 × 10^6^ cells/mL according to microscopic cell counting. A 1-mL volume of the suspension was reserved as an unlabeled blank for flow cytometry while the remaining cells were triturated gently and stained with CM-DiI (Catalog No. V22888; Molecular Probes Inc, Eugene, Oregon; www.probes.com). Briefly, 25 μg/mL CM-DiI staining solution was added to 1 mL of cell suspension and the mixture incubated first at 37 °C under darkness for 5 min and then at 4 °C for 15 min with shaking once every 5 min. Stained cells were centrifuged at 1500 rpm for 5 min, and the supernatant discarded. After rinsing and centrifugation twice in PBS, a fraction of the cells was resuspended at 1 × 10^6^/mL and 1 mL reserved for measuring CM-DiI labeling rate by flow cytometry. The remaining cell suspension was diluted in serum-free medium to 2.5−3 × 10^5^ cells/μL, and stored under darkness in an ice box at 4 °C for subretinal transplantation.

**4. Detection of transplanted hPBMCs in the circulation of rd1 mice**

One month after subretinal injection, 12 mice from each group were anesthetized by intraperitoneal pentobarbital sodium (60 mg/kg body weight) and sacrificed by cervical dislocation. The left eyeball was excised for blood collection. The blood of two randomly selected mice from each group was transferred to prepared anticoagulant tubes containing heparin, mixed well, and then combined 1:1 (v/v) with PBS containing gentamicin. The PBMC fraction was isolated and analyzed as described for human blood samples except that 4−5 mL diluted blood was slowly added to 4−5 mL of lymphocyte separation medium. After centrifugation, the supernatant was removed. Then, 3.5 mL of red blood cell lysate (Beijing Leagene Biotechnology Co. Ltd; China) was added to the centrifuge tube, and the cell suspension was blown repeatedly and carefully for 5 minutes. An appropriate volume of PBS containing gentamicin was added, and the suspension centrifuged at 1600 rpm for 5 min. The supernatant was removed and the pellet resuspended in 10 mL of normal saline. Cell count was determined under a light microscope. After counting, the remaining suspension was centrifuged at 1600 rpm for 5 min, the supernatant discarded, and the pellet resuspended in 2 mL of PBS for immunofluorescence, flow cytometry and quantitative real-time PCR.

The same procedure was repeated on 12 mice in the treatment group at 3 months after cell injection.

**Supplemental Table 1**

**Summary about group sizes of rd1 mice across the entire experiment**

|  |  | control | treated | untreated |  |
| --- | --- | --- | --- | --- | --- |
| Mouse PBMC | Immunofluorescence staining | 4 | 4 | 4 | 1 month |
|  | Flow cytometry | 4 | 4 | 4 |  |
|  | PCR | 4 | 4 | 4 |  |
|  | Immunofluorescence staining |  | 4 |  | 3 month |
|  | Flow cytometry |  | 4 |  |  |
|  | PCR |  | 4 |  |  |
|  | subtotal | 12 | 24 | 12 |  |
|  |  |  |  |  |  |
| Retina | Immunofluorescence staining | 3 | 5 | 3 | 2 week |
|  |  | 3 | 5 | 3 | 1 month |
|  |  | 3 | 5 | 3 | 3 month |
|  | subtotal | 9 | 15 | 9 |  |
|  |  |  |  |  |  |
|  | Flow cytometry | 6 | 8 | 6 | 1 month |
|  |  | 6 | 8 | 6 | 3 month |
|  | subtotal | 12 | 16 | 12 |  |
|  |  |  |  |  |  |
|  | PCR | 6 | 8 | 6 | 1 month |
|  |  | 6 | 8 | 6 | 3 month |
|  | subtotal | 12 | 16 | 12 |  |
|  |  |  |  |  |  |
| total |  | 45 | 71 | 45 | 161 |

**Supplemental Table 2**

**List of antibody that used to Immunofluorescent Staining and Flow Cytometry in pre-induction hPBMCs.**

| Antibody | Antigen | Manufactor | Dilution |
| --- | --- | --- | --- |
| CD3 | T-cell | Invitrogen | 1:250 |
| CD11b | Macrophage | Epitomics | 1:250 |
| CD14 | monocyte | NOVOS | 1:100 |
| CD16 | natural killer cell | Invitrogen | 1:250 |
| CD19 | B-cell | Invitrogen | 1:250 |
| CD34 | hemopoietic stem cell | Abcam | 1:250 |
| CD44 | Memory T cell | Abcam | 1:100 |
| CD45 | leukocyte | Invitrogen | 1:250 |
| vimentin | neural progenitor cell | Abcam | 1:300 |
| βIII-tubulin | neural progenitor cell | Sigma-Aldrich | 1:100 |
| MAP2 | mature neuron | Abcam | 1:250 |
| synapsin | mature neuron | Abcam | 1:250 |
| GFAP | M¨uller glia | Abcam | 1:250 |
| rhodopsin | photoreceptor | Abcam | 1:250 |
| nestin | neural stem cell | Millipore | 1:200 |

S**upplemental Table 3**

**Changes in human peripheral blood mononuclear cell (hPBMC) number after pre-induced culture**

| **Sample** | **Blood volume**  **(mL)** | **Cell number**  **before culture** | **Cell number**  **after culture*** |
| --- | --- | --- | --- |
| 1 | 30 | 3.523×10^7^ | 2.164×10^7^ |
| 2 | 30 | 3.43×10^7^ | 2.873×10^7^ |
| 3 | 30 | 2.826×10^7^ | 1.923×10^7^ |
| 4 | 30 | 2.924×10^7^ | 1.272×10^7^ |
| 5 | 30 | 3.336×10^7^ | 2.526×10^7^ |
| 6 | 30 | 3.279×10^7^ | 2.127×10^7^ |
| Means |  | 3.2219±0.2813×10^7^ | 2.1475±0.5454×10^7^ |

**P=0.0012<0.05** by paired sample t test

*****About two-thirds of the hPBMCs survived after pre-induction for 4 days.

**Supplemental Table 4**

**CM-DiI labeling rate of hPBMCs after pre-induction culture**

|  | **Sample 1** | **Sample 2** | **Sample 3** | **Mean±SD** |
| --- | --- | --- | --- | --- |
| **Blank** | 0.5 | 0.2 | 0.2 | 0.3±0.17 |
| **Labeled cells** | 99.3 | 91.0 | 86.7 | 92.33±6.40 |

**P=0.0015<0.05** by paired sample t tests.

**Supplemental Table 5**

**Lineage marker expression profiles of hPBMCs during pre-induction culture as determined by flow cytometry**

| **Antigen** | **Before induction (% of total, mean** ± SD) | **After induction (% of total, mean** ± SD) | **P value** |
| --- | --- | --- | --- |
| **CD3** | 40.03±5.501 | 27.47±3.329 | 0.0939 |
| **CD11b** | 7.940±0.9627 | 3.100±0.5941 | 0.0064^**^ |
| **CD14** | 1.510±0.5803 | 10.60±3.292 | 0.0303^*^ |
| **CD16** | 10.97±1.801 | 4.140±0.6534 | 0.0348^*^ |
| **CD19** | 12.13±1.650 | 1.567±0.2627 | 0.0060^**^ |
| **CD34** | 3.480±0.7451 | 5.077±0.7574 | 0.1944 |
| **CD44** | 1.350±1.161 | 5.013±0.7801 | 0.0258^*^ |
| **CD45** | 48.33±2.759 | 71.47±5.807 | 0.0380^*^ |
| **GFAP** | 1.553±0.4536 | 2.393±0.5860 | 0.1435 |
| **MAP2** | 2.590±0.1153 | 9.997±2.561 | 0.0402^*^ |
| **Nestin** | 1.047±0.3911 | 5.520±1.265 | 0.0342^*^ |
| **Rhodopsin** | 1.567±0.7729 | 24.99±4.345 | 0.0136^*^ |
| **Synapsin** | 3.457±1.516 | 4.397±1.780 | 0.6346 |
| **Vimentin** | 60.50±2.081 | 98.00±0.5291 | 0.0009^**^ |
| **βⅢ-tubulin** | 1.067±0.5572 | 18.27±0.8327 | 0.0014^**^ |

* means P ＜0.05；** means P ＜0.01

**Supplemental Fig. 1**

**
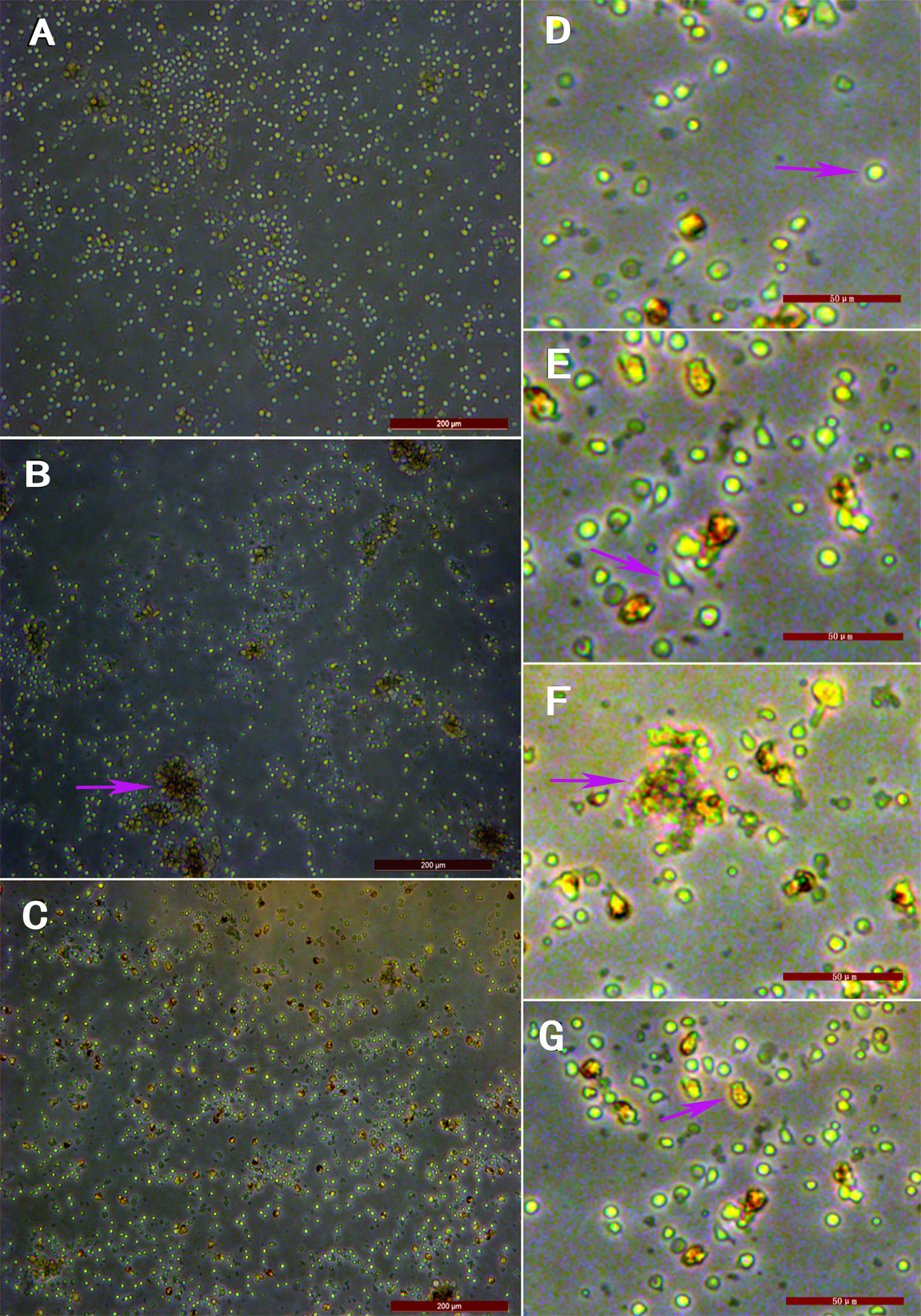
**

**Supplemental Fig. 1: Morphological changes of hPBMCs during pre-induction culture:** Freshly isolated hPBMCs were round, phase-bright, and evenly suspended in the culture medium (A). On Day 1 of pre-induction culture (B), most cells were still suspended in the culture medium individually or in small clusters (indicated by arrow). On Day 3 of pre-induction culture (C), some cells adhered to the wall and exhibit more complex morphology. After four days of pre-induction culture, hPBMCs could be classified into four categories: round cells (D, indicated by arrow), cells with one to three synapses (E, indicated by arrow), cell aggregates (F, indicated by arrow), and large yellow irregular cells (G, indicated by arrow). A−C: 100×. D−G: 400×

**Supplemental Fig. 2.**


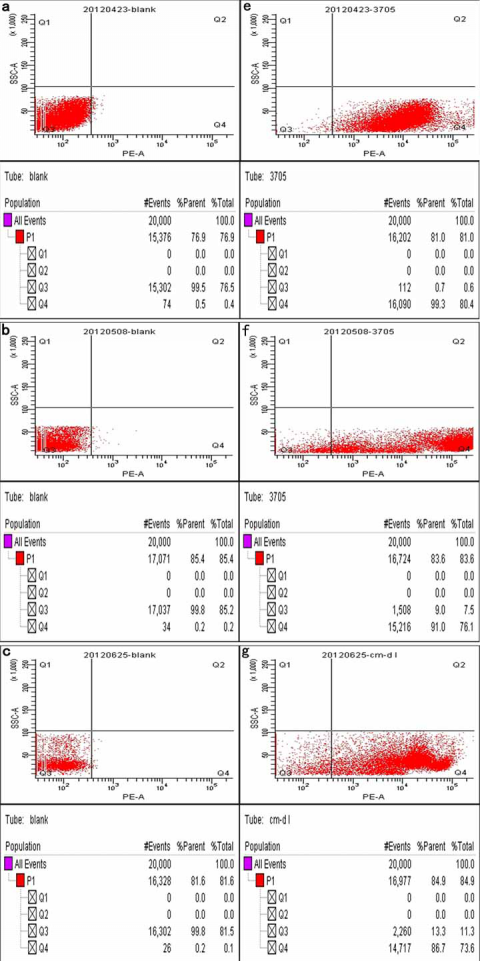


**Supplemental Fig. 2. Counting of CM-DiI-labeled pre-induced hPBMCs by flow cytometry.** (a-c) Blank controls. (e-g) Cell samples. The number of points in zone Q4 relative to the other zones indicates the proportion of CM-DiI-labeled pre-induced hPBMCs. Labeling was 92.33% ± 6.40% efficient.

**Supplemental Fig. 3**


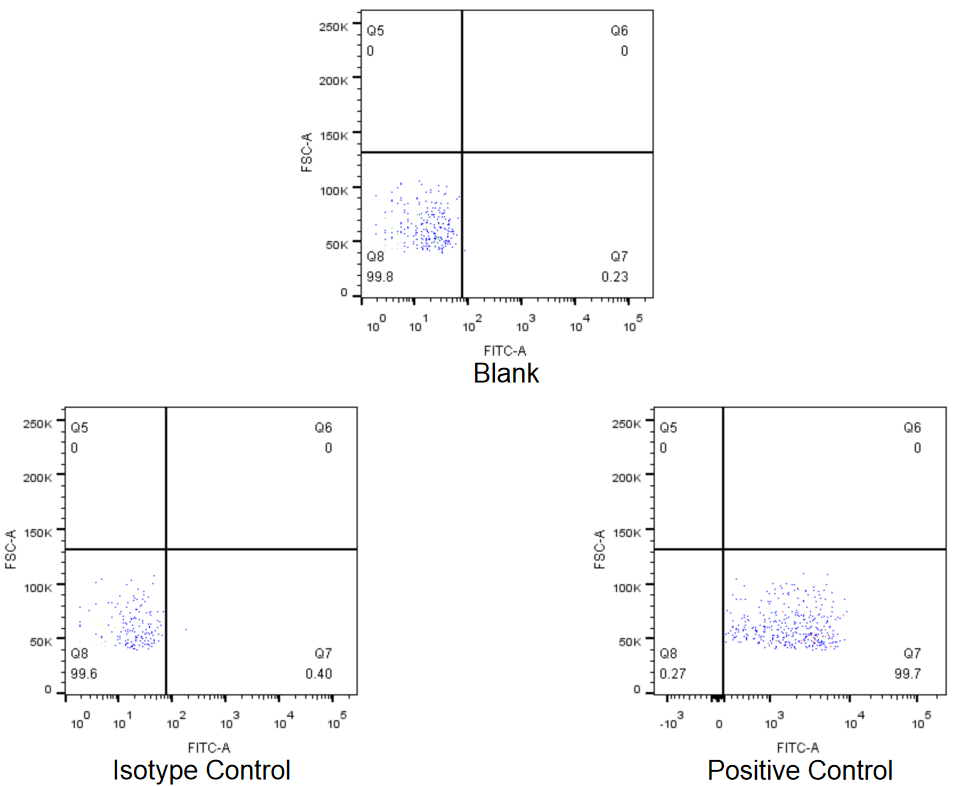


**Supplemental Fig. 3. Flow cytometry of pre-induction hPBMCs labled**

**with human mitochondrial antibody.** Compare to the Blank and Isotype Control, about 99% cells labled with human mitochondrial antibody(Fitc fluorescence).

**Supplemental Fig.4**

**
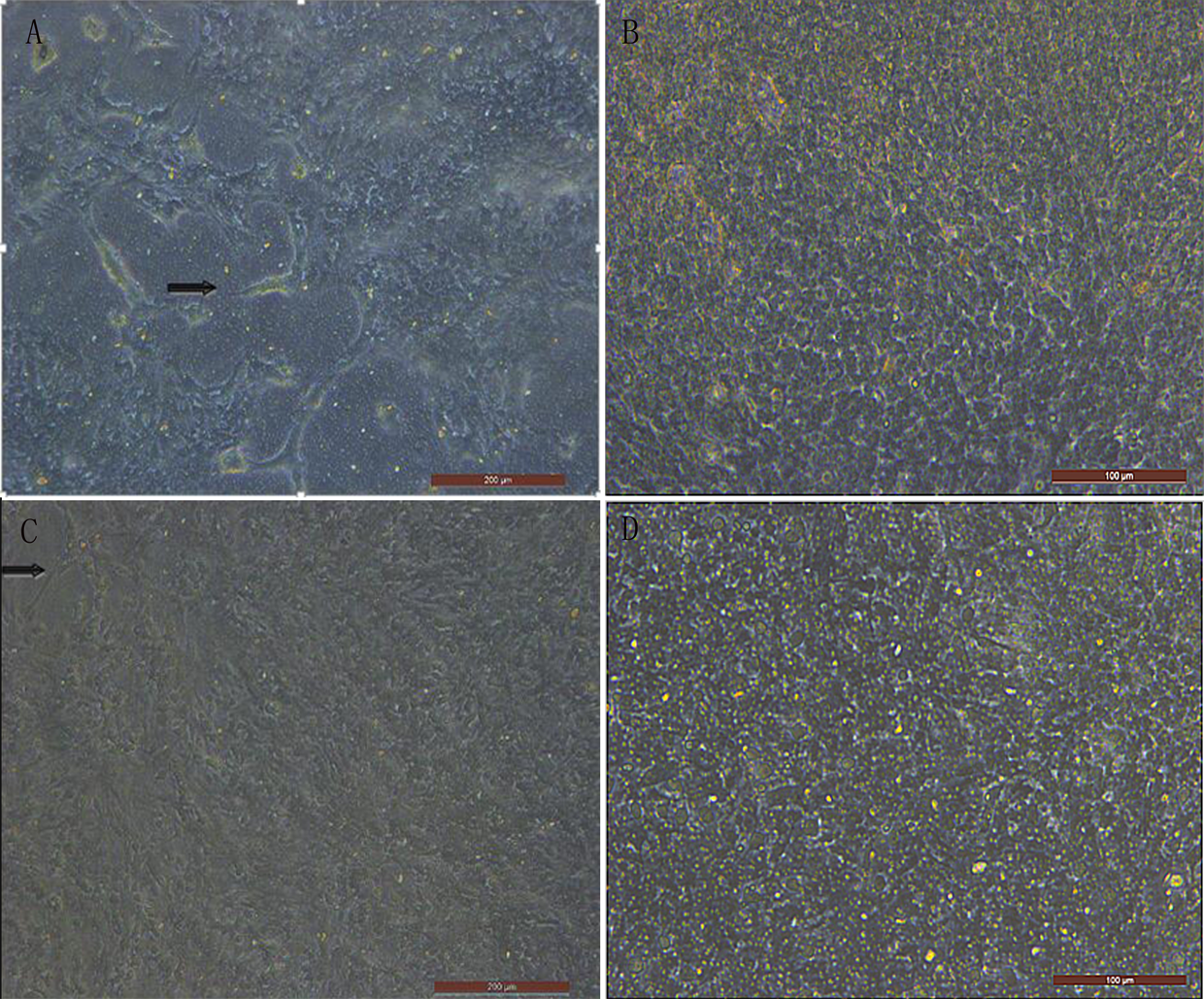
**

**Supplemental Fig. 4. Morphology of rat retinal cells during induction cultivation**. (A) Rat retinal cells after one day of cultivation. By this time, most cells in suspension had adhered to the substrate (in the top chamber of 6-well transwell plates). (B) Retinal cells in the well center after 2–3 days of cultivation. (C) Retinal cells at the well periphery after 2–3 days of cultivation. (D) Retinal cells after four days of cultivation. The arrow indicates a synapse.

**Supplemental Fig. 5**

**

**

**Supplemental Fig. 5. Changes in hPBMC lineage marker expression profile during pre-induction culture as determined by immunofluorescence staining.** The blue fluorescence is from Hoechst nuclear stain while the green fluorescence indicates expression of one of the following retinal or blood lineage markers (as indicated): GFAP, MAP2, nestin, rhodosin, synapsin, vimentin, betaIII-tubulin, CD45, CD14, CD3, CD11b, CD19, CD34, or CD44. During pre-induction culture, expression levels of the neural lineage markers MAP2, nestin, rhodosin, vimentin, beta III-tubulin, CD45, and CD14 increased, while expression of the blood lineage markers CD11b, CD19, and CD16 decreased.

**Supplemental Fig. 6**

**

**

**Supplemental Fig. 6. Immunofluorescence staining of frozen retinal sections from untreated rd1 mice.** Tissue was harvested 2 weeks, 1 month, and 3 months after mice from the same cohort received subretinal injection of serum-free DMEM (control group) or hPBMCs (treatment group)**.** Note the absence of red fluorescence from CM-DiI and green fluorescence from a fluorophore-tagged human mitochondrial antibody (the markers for injected cells). The blue fluorescence is from Hoechst nuclear stain. The Hoechst staining also demonstrates the progressive retinal degeneration characteristic of rd1 mice.

**Supplemental Fig. 7**





**Supplemental Fig. 7. Immunofluorescence staining of frozen retinal sections from control rd1 mice at 2 weeks, 1 month, and 3 months after subretinal serum-free DMEM injection.** Note the absence of CM-DiI fluorescence (red) and human mitochondrial antibody immunostaining (green). The progressive reduction in Hoechst nuclear staining (blue) is indicative of retinal degeneration.

**Supplemental Fig. 8**

**

**

**Supplemental Fig. 8. Flow cytometry results about surviving hPBMCs in 1month retinal cell suspension prepared.** The PE fluorescence(red) is from CM-DiI and the FITC fluorescence(green) is from immunostaining using a human mitochondrial antibody. Zone Q2 is the double labeled area indicative of transplanted hPBMCs.

**Supplemental Fig. 9**





**Supplemental Fig. 9. Flow cytometry results about surviving hPBMCs in 3months retinal cell suspension prepared.** The PE fluorescence(red) is from CM-DiI and the FITC fluorescence(green) is from immunostaining using a human mitochondrial antibody. Zone Q2 is the double labeled area indicative of transplanted hPBMCs.

**Supplemental Fig. 10**





**Supplemental Fig. 10. ERG of C57 mice(n=6).** In the experiment, Each eye was stimulated by both ultraviolet (365nm) and green (505nm) light, and exposed to a 2 ms ultraviolet stimulus intensity of 1.6 log(Cd s/m^2^) and green light stimulus intensity of 1.3 log(Cd s/m^2^) simultaneously. (a-e) Right eyes of C57 mice. (f-j) Left eyes of C57 mice. ( a-wave: -63.13 ± 30.65 μV; b-wave: 203.4 ± 89.1μV)

**Supplemental Fig. 11**

**

**

**Supplemental Fig. 11. ERG of treated mice after 3months(n=20).** In the experiment, Each eye was stimulated by both ultraviolet (365nm) and green (505nm) light, and exposed to a 2 ms ultraviolet stimulus intensity of 1.6 log(Cd s/m^2^) and green light stimulus intensity of 1.3 log(Cd s/m^2^) simultaneously. (a-e) Right eyes of treated mice. (f-j) Left eyes of treated mice. (a-wave: right eyes = -3.71±3.56 μV, left eyes = -2.83 ± 2.68 μV; b-wave: right eyes = 25.67 ± 12.53 μV, left eyes = 21.47 ± 13.19 μV).

**Supplemental Fig. 12**

**

**

**Supplemental Fig. 12**. **Immunofluorescence staining of mouse PBMCs from the untreated group, control group, and treatment group at 1 and 3 months after transplantation.** There were no CM-DiI-positive cells (red) or human mitochondrial antibody positive cells (green), only Hoechst staining (blue).
